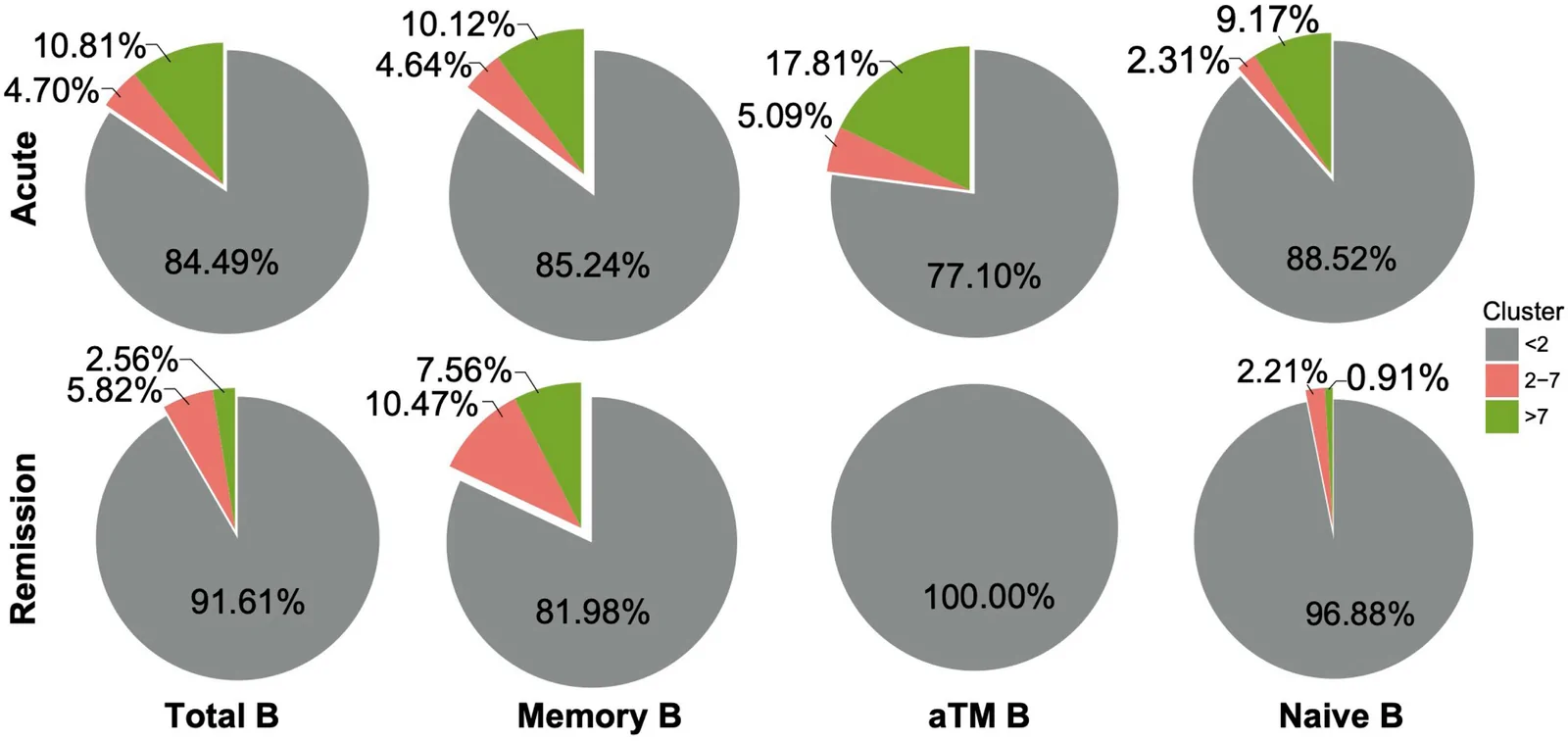# *LAMA5* pathogenic variant uncovers a novel autoantigen in membranous nephropathy

**DOI:** 10.1016/j.gendis.2026.102301

**Published:** 2026-06-13

**Authors:** Han Xiao, Hui Yin, Yulu Shi, Xindi Zhou, Qinqin Tan, Lan Qiu, Chenyang Xiong, Wanbing Chen, Xuejun Yang, Wei Jiang, Haiping Yang, Qiu Li

**Affiliations:** Department of Nephrology, Children's Hospital of Chongqing Medical University, National Clinical Research Center for Children and Adolescents' Health and Diseases, Ministry of Education Key Laboratory of Child Development and Disorders, Chongqing Key Laboratory of Pediatric Metabolism and Inflammatory Diseases, Chongqing 401122, China

**Keywords:** Autoantigen, Laminin α5, Membranous nephropathy, Nephrotic syndrome, Rituximab

## Abstract

Membranous nephropathy is a leading cause of nephrotic syndrome, driven by autoantibodies targeting podocyte antigens. Although antibodies against PLA_2_R and THSD7A account for the majority of cases, a substantial fraction of patients remain seronegative, implying the existence of additional, unidentified autoantigens. Here, we report the identification of two novel compound heterozygous mutations in *LAMA5* encoding Laminin α5, in a pediatric patient with severe nephrotic syndrome. Whole-exome sequencing revealed c.1355A>T (p.N452I) and c.9770A>G (p.N3257S) variants, with structural modeling indicating that p.N452I induces a conformational change in Laminin α5. This altered conformation enhanced its binding to Collagen IV networks, resulting in thickening of the glomerular basement membrane and the creation of a neo-epitope recognized by conformation-specific IgG1/IgG3 autoantibodies. These autoantibodies activated the classical complement pathway, triggering podocyte injury. A knock-in mouse model harboring the *Lama5*^*N457I*^ mutation recapitulated the human phenotype, exhibiting proteinuria, glomerular basement membrane thickening, and glomerular IgG deposits. Notably, rituximab treatment in the patient led to the disappearance of anti-Laminin α5 autoantibodies and sustained clinical remission. Our findings establish Laminin α5 as a novel genetic autoantigen in membranous nephropathy and suggest that screening for anti-Laminin α5 antibodies may help identify patients who could benefit from B-cell-targeted therapies.

## Introduction

The glomerular basement membrane (GBM) is a specialized extracellular matrix essential for maintaining the structural and functional integrity of the glomerular filtration barrier. Its core components, including Laminin α5β2γ1, Collagen IV α3α4α5 networks, and integrin α3β1, orchestrate both architectural stability and cell–matrix signaling critical for podocyte adhesion and function.^1^ Deviations or irregularities in these integral components have the potential to induce structural anomalies within the GBM. This intricate interplay significantly influences the morphology and functionality of podocytes, as exemplified by conditions such as thin basement membrane nephropathy and Alport syndrome.^1^, ^2^, ^3^ In addition to these structural considerations, immune glomerular diseases encompass a range of disorders resulting from the accumulation of immune complexes within the glomeruli. Within this category, a notable subset comprises GBM-associated diseases, including conditions such as IgA nephropathy, membranous proliferative glomerulonephritis, and membranous nephropathy (MN). Despite the recognition of these associations, the precise relationship between immune complex deposition in glomeruli and GBM lesions remains uncertain.^4^, ^5^, ^6^ Further research is needed to elucidate the intricate connections between immune-mediated mechanisms and GBM-related pathology in glomerular diseases.

MN is associated with significant antigens such as PLA_2_R and THSD7A, and recent research has unveiled novel antigens like Netrin G1 and protocadherin FAT1.^7^^,^^8^ However, identifying a definitive target antigen remains challenging, as not all MN cases are linked to known antigens, suggesting the involvement of unidentified factors. Importantly, genetics plays a pivotal role in MN development, as evidenced by studies linking genetic variations, like *PLA2R1* and *HLA-DQA1* allele, to MN susceptibility.^9^, ^10^, ^11^ This underscores the intricate interplay between genetics and MN, contributing significantly to our comprehension of the disease and holding promise for enhanced diagnostics and treatment modalities.

Here, we identify a novel disease mechanism in which compound heterozygous mutations in *LAMA5* (c.1355A>T, p.N452I, and c.9770A>G, p.N3257S), encoding the Laminin α5 subunit, drive autoimmune glomerular injury. Unlike COL4A3/4/5-related diseases, the novel *LAMA5* variants induce conformational changes in Laminin α5 that lead to its overexpression, enhanced binding to Collagen IV α3/α4/α5, and pathological GBM thickening. Critically, the p.N452I mutation generates a neo-epitope that elicits conformation-dependent, IgG1/IgG3-dominant autoantibodies specifically targeting Laminin α5. These antibodies deposit in the GBM, activate the classical complement pathway through C1q, C3b, and C5b, and mediate podocyte injury, consistent with secondary MN. Notably, this autoimmune response is responsive to B-cell depletion therapy that anti-Laminin α5 autoantibodies disappear following rituximab treatment and correlate with clinical remission, suggesting their utility as a biomarker for patient stratification. These findings redefine the role of Laminin α5 beyond structural support, positioning it as a genetically determined autoantigen in a distinct subset of MN. This work bridges genetic etiology and immune pathogenesis, offering a mechanistic framework for precision diagnostics and targeted immunotherapy in pediatric MN.

## Materials and methods

### Clinical data and sample collection

Demographic and clinical data were comprehensively collected from patients' medical records, including age, gender, clinical presentation, laboratory results, and treatment plans. The study included an observational cohort, diagnosed between 2016 and 2025, with pathogenic/likely pathogenic variants involved in GBM-associated genes and available renal biopsy reports. Diagnosis of Alport syndrome in individuals with persistent hematuria and/or proteinuria should fulfill at least one of these criteria.^12^, ^13^, ^14^ Individuals with persistent isolated glomerular hematuria and diffuse thin GBM (< 250 nm) were defined as thin basement membrane nephropathy.^15^ Mesangial proliferative glomerulonephritis, minimal change disease, and IgA nephropathy were confirmed by histological diagnoses.^16^, ^17^, ^18^ Diagnosing MN in individuals under 18 necessitated a thorough clinical evaluation to rule out other possible underlying causes. The diagnosis was confirmed through a kidney biopsy and further supported by the detection of anti-PLA_2_R or anti-THSD7 antibodies. Healthy controls matched for age and sex were selected from clinically healthy outpatient subjects without a history of renal or autoimmune diseases. Ethical approval (No. 2023-304) was obtained from the Children's Hospital of Chongqing Medical University Ethics Committee, aligning with the principles of the Declaration of Helsinki for ethical research conduct.

### Exome sequencing

Genomic DNA was extracted from EDTA-treated peripheral blood samples using QIAamp DNA Blood Mini kits (QIAGEN Science, Germantown, Maryland, USA). Subsequently, we conducted high-throughput whole-genome sequencing with PE150 sequencing on an Illumina NovaSeq6000 sequencer at the Joy Orient Translational Medicine Research Center in Beijing. The target region showed 96.7% coverage at > 10 × depth, 93.9% at > 20 × depth, and 90.4% at > 30 × depth. Quality control was carried out using Fastp software, and all identified variants were validated through Sanger sequencing. Pathogenicity assessment followed the guidelines of the American College of Medical Genetics and Genomics (ACMG). Protein domains analysis was performed using the Simple Modular Architecture Research Tool (SMART). Multiple sequence alignment was conducted using Clustal Omega. Protein domain crystal structures analysis was carried out using SWISS-MODEL.

### Confocal and fluorescence microscopy

Kidney sections and HeLa cells were fixed using neutral buffered formalin and subsequently embedded using standard procedures into either paraffin or optimal cutting temperature compound. To counteract nonspecific binding, a 10% goat serum was employed at room temperature for 90 min. Following this, samples were incubated at 4 °C overnight with primary antibodies within a blocking buffer. Fluorochrome-conjugated secondary antibodies (Invitrogen, USA) were utilized for visualizing staining, while DAPI (1:1000, C1002, Beyotime, China) was employed to stain DNA. Capturing of optical images occurred using a Nikon A1R Meta confocal microscope. The primary antibodies used were list below: Laminin α5 (1: 200, ab210957, Abcam, UK), Nephrin (1:200, GP-N2, Progen, German), Podocin (1:200, PA5-79757, Invitrogen, USA), Synaptopodin (1:200, 21064-1-AP, Proteintech, China), WT1 (1:100, YM6533, Immunoway, USA), COL4A3 (1:100, #7076, Chondrex, USA), COL4A4 (1:100, #7073, Chondrex, USA), COL4A5 (1:100, #7077, Chondrex, USA), Integrin β1 (1:100, #34971S, Cell Signaling Technology, USA), Laminin β2 (1:200, ab210956, Abcam, UK), Laminin γ1 (1:200, ab233389, Abcam, UK), Flag (1:1000, F1804, Sigma–Aldrich, German), PLA_2_R (1:500, ab211490, Abcam, UK), IgG (1:800, ab109489, Abcam, UK), IgG1 (1:1000, RS010215, Immunoway, USA), IgG2 (1:100, 9080-01, SouthernBiotech, USA), IgG3 (1:1000, 9210-01, SouthernBiotech, USA), IgG4 (1:800, NBP2-62126, Novus Biologicals, USA), C5b-9 (1:1000, ab55811, Abcam, UK), C3b (1:100, ab11871, Abcam, UK), C1q (1:100, ab11861, Abcam, UK), MBL (1:400, ab190834, Abcam, UK), CFB (1:200, ab309149, Abcam, UK).

### Immunohistochemistry

Immunohistochemical staining was conducted by first deparaffinizing the paraffin sections, followed by rehydration. Heat-induced antigen retrieval buffer was applied for effective pretreatment, while a peroxidase-blocking reagent (ZSJB-BIO, Beijing, China) was utilized for a 10-min blocking step. Subsequently, the sections underwent incubation with primary antibodies targeting Laminin α5 (1:200, ab210957, Abcam, UK) and COL4A5 (1:50, #7077, Chondrex, USA) at 4 °C overnight. Bound antibodies were detected using the diaminobenzidine system (DA1010, Solarbio, China). Digital images were captured using an Olympus light microscope, and the Image-Pro Plus 6.0 software facilitated subsequent image analysis.

### Cells and mice

HeLa and 293T cells were maintained in Dulbecco's modified Eagle medium supplemented with 10% (v/v) fetal bovine serum and 1% (v/v) penicillin/streptomycin at 37 °C in a humidified 5% CO_2_ atmosphere. Cells were seeded into six-well culture plates at a density of 1.5 × 10^5^ cells/well for 24 h before transfection. The plasmid pEnCMV-*LAMA5* (Human)-3 × FLAG-SV40-Neo was sourced from the MiaoLing Plasmid Sharing Platform (MLPSP, China) and validated by Sanger sequencing. Site-directed mutagenesis of *LAMA5* was performed using the Mut Express MultiS Fast Mutagenesis Kit V2 (Vazyme, C215), followed by confirmation through Sanger sequencing. Transfections were carried out using Lipofectamine 3000 (ThermoFisher, USA) according to the manufacturer's protocol, with 2500 ng of plasmid DNA per well. The medium was refreshed 6–7 h post-transfection.

To generate the *Lama5*^*N457I*^ point mutation mice, CRISPR/Cas9 technology was employed by Cyagen Biosciences (ShuZhou, China), targeting the human c.1355A>T (p.N452I) mutation site. Balb/C mice served as the genetic background. After several generations, homozygous mutants and wild-type littermate controls were selected for further analysis. Urine samples were collected randomly and analyzed for protein levels via SDS-PAGE followed by Coomassie Brilliant Blue staining. At 6–8 weeks of age, mice were deeply anesthetized with tribromoethanol (Avertin) and perfused intracardially with cold phosphate-buffered saline (PBS). Kidneys were harvested, with some lysed for total protein extraction and others fixed in 4% paraformaldehyde before paraffin embedding. All animal procedures were approved by the Ethics Committee of the Children's Hospital of Chongqing Medical University.

### Co-immunoprecipitation assay and Western blotting

Cells were harvested and lysed using cell lysis buffer (P0013, Beyotime, China), with added protease inhibitors. Protein concentration was quantified using the BCA protein assay (MA0082, Meilunbio, China). Equal quantities of total protein were then incubated at 4 °C overnight with Anti-Flag beads (B26101, Bimake, USA), each in its respective experimental group. On a subsequent day, the beads were subjected to three washes with PBS-Tween 20 buffer, and purified proteins were extracted by boiling them in SDS-PAGE loading buffer (P0015A, Beyotime, China). For Western blotting analysis, cell samples were extracted, quantified, and then boiled at 95 °C for 10 min. Protein samples were separated on a 6% sodium dodecyl sulfate-polyacrylamide gel electrophoresis gel and subsequently transferred to a polyvinylidene fluoride (PVDF) membrane using established protocols. Subsequently, membranes were blocked with 5% non-fat dry milk in Tris-buffered saline with Tween-20. Primary antibodies, including Flag (1:3000, F1804, Sigma–Aldrich, Germany), COL4A3 (1:1000, A16359, Abclonal, China), COL4A4 (1:1000, 19674-1-AP, Proteintech, China), and COL4A5 (1:1000, 05005, BiCell Scientific, USA), were incubated at 4 °C overnight. Following washing, blots were exposed to horseradish enzyme-labeled goat anti-rabbit or goat anti-mouse IgG (1:1000, ZSGB-BIO, China) at room temperature for 1 h. Subsequently, an enhanced chemiluminescence assay (32209, ThermoFisher, USA) was employed to visualize bands, and the resulting images were analyzed using ImageJ software.

### Protein G immunoprecipitation procedure for autoantibody detection

For protein G immunoprecipitation, 293T cells were lysed using RIPA lysis buffer within 24–48 h post-transfection, followed by quantification of protein concentration using the BCA assay. A mixture containing 500 μg of lysed cell protein and 50 μL of plasma samples collected during remission, active disease intervals, and from healthy controls was created. This mixture underwent overnight incubation at 4 °C. Subsequently, 75 μL (equivalent to 2.1 mg) of pre-washed Dynabeads Protein G (10004D, Invitrogen, USA) were added to the protein-plasma premix. The sample was then subjected to 120 min of rotation at 4 °C. After this, a magnet was employed to facilitate the removal of supernatant. The samples were then subjected to three washes with PBS-Tween 20 buffer (200 μL PBS with 0.02% Tween 20) to reduce nonspecific binding interactions. To elute immunoprecipitated IgG-antigen complexes, the Dynabeads were subjected to boiling in 1 × SDS-PAGE loading buffer for 5 min. Subsequent Western blotting analysis was performed to detect Laminin α5 within the eluted protein.

### Single-cell RNA sequencing and data analysis

Cryopreserved peripheral blood mononuclear cells were thawed and subjected to fluorescence-activated cell sorting (BD Biosciences). Single-cell RNA sequencing and V(D)J libraries were generated using the 10 × Genomics Chromium platform. Library concentrations were quantified with the Qubit High Sensitivity DNA Assay (Thermo Fisher Scientific), and fragment sizes were assessed on a Bioanalyzer 2200 (Agilent). Sequencing was performed on the DNBSEQ-T7 platform (MGI) using 150 bp paired-end reads. Raw sequencing reads were preprocessed with fastp and aligned to the human reference genome (GRCh38) using Cell Ranger (10x Genomics). Cells were retained if they expressed > 200 genes and exhibited < 10% mitochondrial UMI content. Downstream analysis was conducted in Seurat (v4.1.1): data were normalized (LogNormalize), scaled, and subjected to principal component analysis based on the top 2000 highly variable genes. The first 10 principal components were used for dimensionality reduction and visualization via t-distributed Stochastic Neighbor Embedding (t-SNE) and Uniform Manifold Approximation and Projection (UMAP).

Cluster-specific marker genes and differentially expressed genes were identified using the Wilcoxon rank-sum test (FindMarkers) with thresholds of |log_2_ fold change| > 0.25, *p* < 0.05, and min.pct > 0.1. Gene Set Enrichment Analysis (GSEA) was performed using Fisher's exact test, with *p*-values adjusted for multiple testing via the Benjamini–Hochberg procedure. Enriched annotations included Gene Ontology (GO), Kyoto Encyclopedia of Genes and Genomes (KEGG), Hallmark pathways, chromosomal location gene sets, and a custom 41-gene signature derived from CellPhoneDB. Additionally, QuSAGE was applied to evaluate gene set activity at the single-cell level using a domestic gene set and the normalized expression matrix.

Cell–cell communication networks were inferred using the CellChat R package with the CellChatDB.human database, assuming shared cell types across samples. For single-cell B cell receptor sequencing, V(D)J sequences were processed with Cell Ranger, and clonotypes were defined in Scirpy (v0.11.2) based on CDR3 nucleotide sequence identity and V gene usage.

### Statistical analysis

Data analysis was carried out utilizing the two-tailed Student's *t*-test in GraphPad Prism 10 software. The results were presented as mean ± standard error of the mean. Significant differences were determined at ∗*p* < 0.05, ∗∗*p* < 0.01, and ∗∗∗*p* < 0.001. Each experiment was conducted with a minimum of three independent samples.

## Results

### *COL4A3/4/5* mutations in Alport syndrome alter GBM structure without inducing IgG immune complex deposition

To investigate whether pathogenic variants in GBM-associated genes are linked to IgG immune complex deposition, we retrospectively analyzed clinical cases with confirmed pathogenic or likely pathogenic variants in GBM-related genes at the Children's Hospital of Chongqing Medical University between 2016 and 2025. Using an integrated screening workflow that combined genetic data with renal biopsy reports categorized by the presence or absence of glomerular IgG deposits, we assembled a cohort of 97 patients diagnosed with Alport syndrome, thin basement membrane nephropathy, IgA nephropathy, or other glomerulopathies (Fig. 1 and Table 1). Focusing on Alport syndrome caused by mutations in *COL4A3*, *COL4A4*, or *COL4A5*, we selected three representative cases for detailed histopathological and ultrastructural evaluation. Light microscopy (hematoxylin–eosin, PAS, Masson, PASM staining) and transmission electron microscopy revealed hallmark GBM abnormalities, including lamellation and thickening, but no detectable IgG immune complex deposition (Table 2 and Fig. S1). Immunofluorescence demonstrated partial loss of linear expression of the Collagen IV α3α4α5 network in the GBM corresponding to the mutated genes (Fig. 2A and B). Concurrently, expression of Laminin subunits α5, β2, and γ1 was reduced (Fig. 2B and C), whereas Integrin β1 exhibited preserved linear staining across all three cases (Fig. 2B). These findings indicate that mutations in *COL4A3/4/5* disrupt GBM structural integrity but do not induce autoimmune IgG complex formation or deposition.

**Figure 1:**
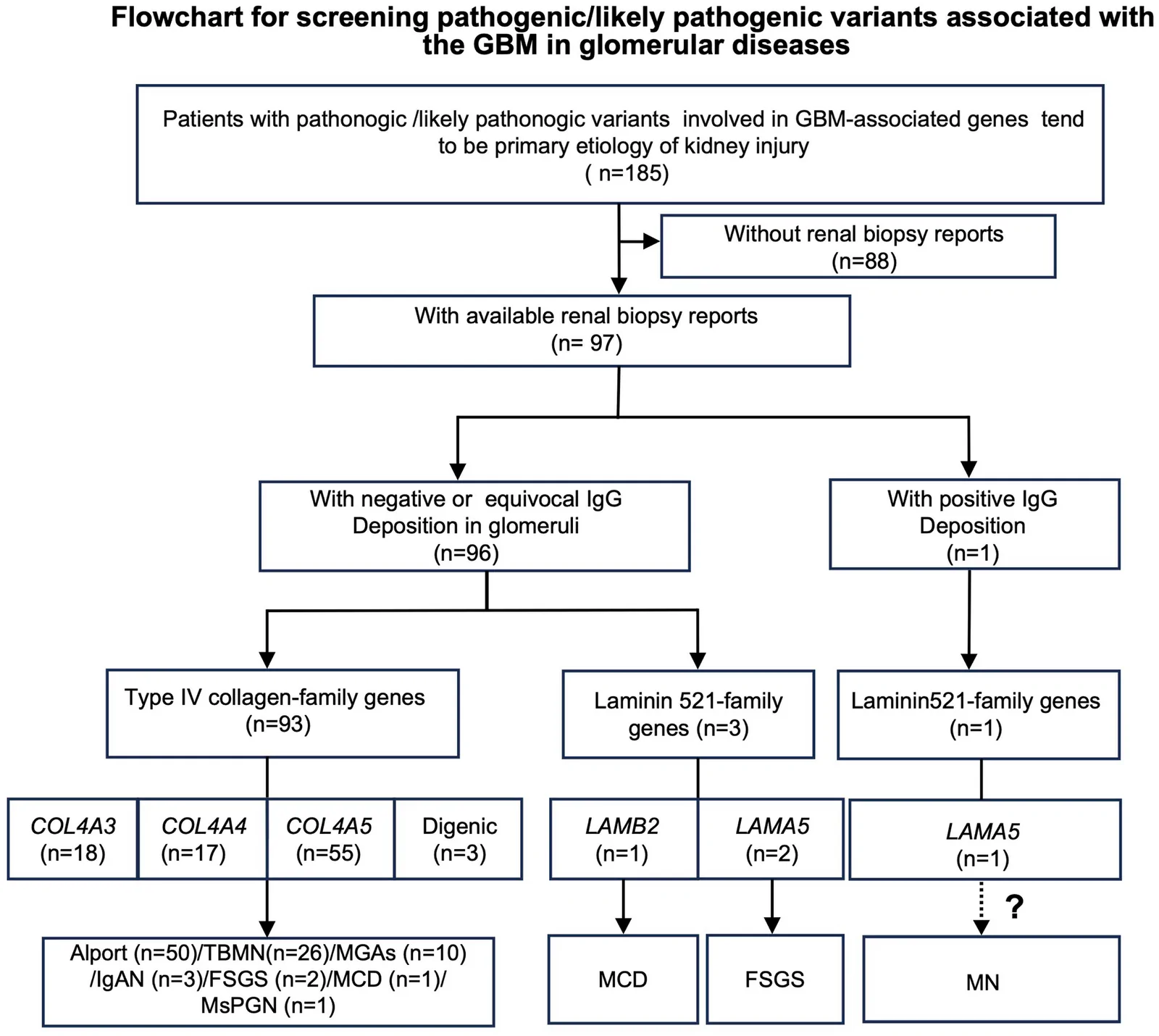
Flowchart detailing the collection and analysis of clinical patients with pathogenic/likely pathogenic variants in genes associated with the glomerular basement membrane (GBM). Patients harboring such variants in GBM-related genes but lacking accompanying renal biopsy reports (*n* = 88) were excluded. The analysis concentrated on individuals with available renal biopsy reports (*n* = 97), and they were categorized based on the presence or absence of IgG immune complexes in glomeruli. The deposition patterns were then stratified into two distinct groups. The group with negative or equivocal glomerular IgG immune complex deposition screened 93 cases of GBM-involved kidney disease caused by *COL4A3/4/5* gene mutations and 3 cases of MCD and FSGS caused by *LAMB2* and *LAMA5* gene mutations, respectively. In contrast, the group with glomerular IgG immune complex deposition identified one case of *LAMA5* mutation in this study, which may lead to membranous nephropathy (MN). MsPGN, mesangial proliferative glomerulonephritis; TBMN, thin basement membrane nephropathy; FSGS, focal segmental glomerulosclerosis; MCD, minimal change disease; IgAN, immunoglobulin A nephropathy; MGAs, minor glomerular abnormalities.

**Table 1:** Clinical data of the patients with pathogenic/likely pathogenic variants involved in glomerular basement membrane-associated genes.

Characteristics	COL4A3 (*n* = 18)	COL4A4 (*n* = 17)	COL4A5 (*n* = 55)	Digenic (*n* = 3)
**Clinical characteristics**				
Age (yr), median (IQR)	7.33 (3.87, 10.27)	6.00 (5.42, 8.25)	4.50 (2.50,7.00)	6.66 (6.00,13.00)
Male sex, *n* (%)	8 (44.44)	13 (76.47)	29 (52.73)	1 (33.33)
Time to biopsy (mo), median (IQR)	13.50 (6.48, 36.21)	17.04 (4.50, 53.52)	13.08 (3.04,40.08)	0.96 (0.72, 2.04)
Hematuria, *n* (%)	18 (100.00)	18 (100.00)	55 (100.00)	3 (100.00)
Proteinuria (24 h UPE >150 mg/24 h), n (%)	8 (44.44)	8 (44.44)	40 (70.91)	2 (66.67)
eGFR (mL/min per 1.73 m^2^), median (IQR)	123.46 (101.38,127.60)	126.62 (98.07,134.67)	117.00 (101.02,131.59)	127.60 (109.63,131.49)
eGFR < 90 mL/min per 1.73 m2, *n* (%)	3 (16.67)	3 (16.67)	8 (44.44)	0 (0)
cIgG (g/L), median (IQR)	9.29 (7.24,11.38)	10.50 (7.46,12.60)	8.02 (6.02,10.30)	10.10 (8.76, 15.30)
cIgA (g/L), median (IQR)	0.98 (0.71,1.58)	1.33 (0.98,1.96)	1.09 (0.66,1.40)	1.59 (1.06, 2.57)
cIgM (g/L), median (IQR)	1.14 (0.89,1.56)	1.18 (1.06, 1.45)	1.35 (1.03,1.66)	1.82 (1.02,2.61)
cIgE (IU/mL), median (IQR)	33.75 (12.65,92.08)	74.00 (31.85, 119.60)	36.80 (15.55,159.00)	33.60 (26.90,123.60)
cC3, median (IQR)	0.88 (0.74,1.13)	0.90 (0.70,1.02)	0.87 (0.74,1.03)	0.98 (0.70,0.99)
cC4, median (IQR)	0.19 (0.16,0.24)	0.17 (0.14,0.19)	0.18 (0.14,0.24)	0.20 (0.12,0.22)
**Immunoglobulin or complement deposition**				
IgG (−/+/1+/2+/3+), *n* (%)	16/2/0/0/0 (88.89/11.11/0/0/0)	17/0/0/0/0 (100/0/0/0/0)	54/1/0/0/0 (98.18/1.82/0/0/0)	3/0/0/0/0 (100/0/0/0/0)
IgA (−/+/1+/2+/3+), *n* (%)	16/2/0/0/0 (94.12/11.11/0/0/0)	14/1/0/1/1 (85.00/5.00/0/5.00/5.00)	44/8/1/1/1 (82.14/14.04/1.75/1.75/1.75)	3/0/0/0/0 (100/0/0/0/0)
IgM (−/+/1+/2+/3+), *n* (%)	1/6/11/0/0 (5.56/33.33/61.11/0/0)	1/3/13/0/0 (5.88/17.65/76.47/0/0)	2/10/42/0/1 (3.64/18.18/76.36/0/1.82)	1/0/2/0/0 (33.33/0/66.67/0/0)
C3 (−/+/1+/2+/3+), *n* (%)	16/2/0/0/0 (88.89/11.11/0/0/0)	15/2/0/0/0 (88.24/11.76/0/0/0)	47/5/2/1/0 (85.45/9.09/3.64/1.82/0)	3/0/0/0/0 (100/0/0/0/0)
C1q (−/+/1+/2+/3+), *n* (%)	16/2/0/0/0 (88.89/11.11/0/0/0)	16/1/0/0/0 (94.12/5.88/0/0/0)	50/5/0/0/0 (90.91/9.09/0/0/0)	3/0/0/0/0 (100/0/0/0/0)
**GBM ultrastructural features**				
Diffuse or segmental thickening (> 450 nm), *n* (%)	0 (0)	0 (0)	5 (9.09)	0 (0)
Irregular thickening and thinking (250 nm–450 nm or coexistence of thickening and thinking), *n* (%)	2 (11.11)	1 (5.88)	15 (27.27)	1 (33.33)
Diffused or segmental thinking (< 250 nm), *n* (%)	16 (88.89)	16 (94.12)	35 (63.64)	2 (66.67)
Lamination or splitting or basket weave pattern, *n* (%)	4 (22.22)	3 (17.65)	28 (50.91)	2 (66.67)
Diffused foot process fusion, *n* (%)	2 (11.11)	3 (17.65)	5 (9.09)	0 (0)
Mesangial hypercellularity and/or increase in mesangial matrix, *n* (%)	17 (94.44)	16 (94.12)	50 (90.91)	3 (100.00)
**Mutation types**				
Missense, *n* (%)	14 (66.67)	11 (57.89)	34 (54.84)	4 (66.67)
Nonsense, *n* (%)	1 (4.76)	2 (10.53)	7 (11.29)	0 (0)
Frameshift, *n* (%)	4 (19.05)	2 (10.53)	6 (9.68)	0 (0)
Splice site, *n* (%)	2 (9.52)	4 (21.05)	9 (14.52)	1 (16.67)
Exon deletion, *n* (%)	0 (0)	0 (0)	3 (4.84)	1 (33.33)
Exon duplication, *n* (%)	0 (0)	0 (0)	3 (4.84)	0 (0)

**Table 2:** Genetic variant information for index patients with Alport syndrome.

Index Patient	Gene	Chromosome position	Transcript	Exon	Base (Amino Acid)	Function	Type of mutation	Inheritance
A3-1	*COL4A3*	chr2:228029481-228029505; chr2:228124517	NM_000091; NM_000091	Exon1; Exon19	c.40_63delCTGCCGCTCCTGCTGGTGCTCCTG (p.L14_L21del); c.1038T>A (p.Y346X)	Deletion; Missense	Germiline	Het
A3-2	*COL4A3*	chr2:228145695; chr2:228175529	NM_000091; NM_000091	Exon31; Exon51	c.2461G>T (p.G821C); c.4793T>G (p.L1598R)	Missense; Missense	Germiline	Het
A3-3	*COL4A3*	chr2:228137824	NM_000091	Exon26	c.1918G>A (p.G640R)	Missense	Germiline	Het
A4-1	*COL4A4*	chr2:227917111	NM_000092.5	Exon32	c.2878G>A (p.G960R)	Missense	Germiline	Het
A4-2	*COL4A4*	chr2:227942741	NM_000092	Exon25	c.1856G>A (p.G619D)	Missense	Germiline	Het
A4-3	*COL4A4*	chr2:227942777	NM_000092	Exon25	c.1820C>T (p.A607V)	Missense	Germiline	Het
A5-1	*COL4A5*	chrX:107845150	NM_000495	Exon27	c.2077G>A (p.G693R)	Missense	Germiline	Het
A5-2	*COL4A5*	chrX:107829929	NM_000495	Exon19	c.1117C>T (p.R373X)	Missense	Germiline	Het
A5-3	*COL4A5*	chrX:107846266	NM_000495.5	Exon28	c.2219G>A (p.G740E)	Missense	Germiline	Het

**Figure 2:**
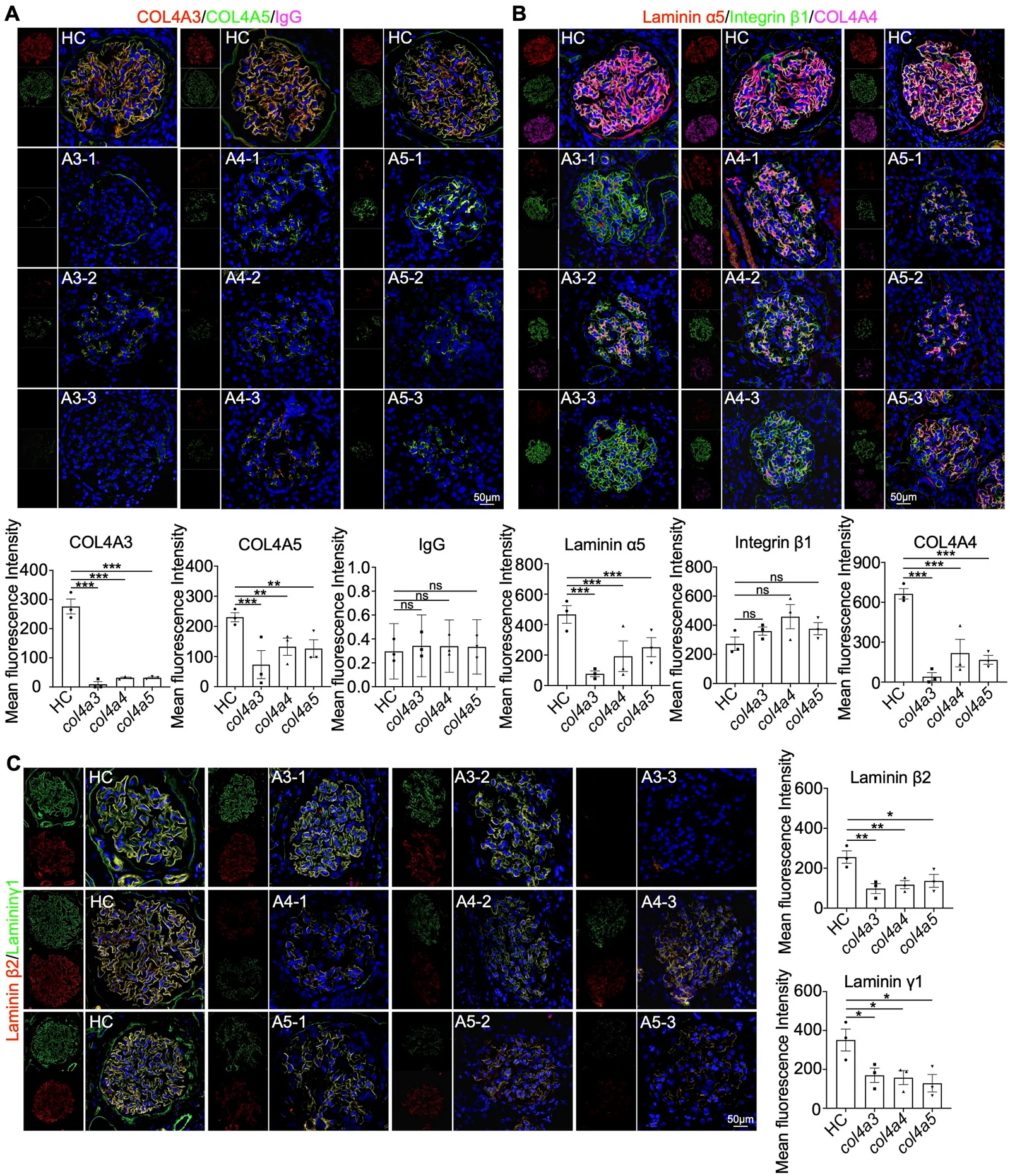
Immunofluorescence staining revealed aberrant expression of the linear structures for glomerular basement membrane (GBM)-related components, including COL4A3/4/5, Laminin α5β2γ1, and Integrin β1, with no observed IgG deposition in individuals with Alport syndrome induced by mutations in *COL4A3, COL4A4*, and *COL4A5* genes, respectively. **(A)** Three-label immunofluorescence comparative analysis was conducted to investigate the correlation between the linear structural expression of COL4A3 and COL4A5 and IgG deposition in GBM of Alport syndrome induced by mutations of *COL4A3*/4/*5*, respectively. **(B)** Another three-label immunofluorescence comparative analysis examined the linear structural expression of Laminin α5, Integrin β1, and COL4A4 in GBM of Alport syndrome caused by mutations in *COL4A3/4/5*. **(C)** Double-label immunofluorescence analysis was conducted to investigate the linear structural expression of Laminin β2 and Laminin γ1 in GBM of Alport syndrome caused by mutations in *COL4A3/4/5* genes. HC, health control. ns, no significant; ∗*p* < 0.05, ∗∗*p* < 0.01, and ∗∗∗*p* < 0.001.

### The novel compound heterozygous *LAMA5* mutations are associated with laminin α5 dysregulation and autoimmune glomerular injury

**Figure 3:**
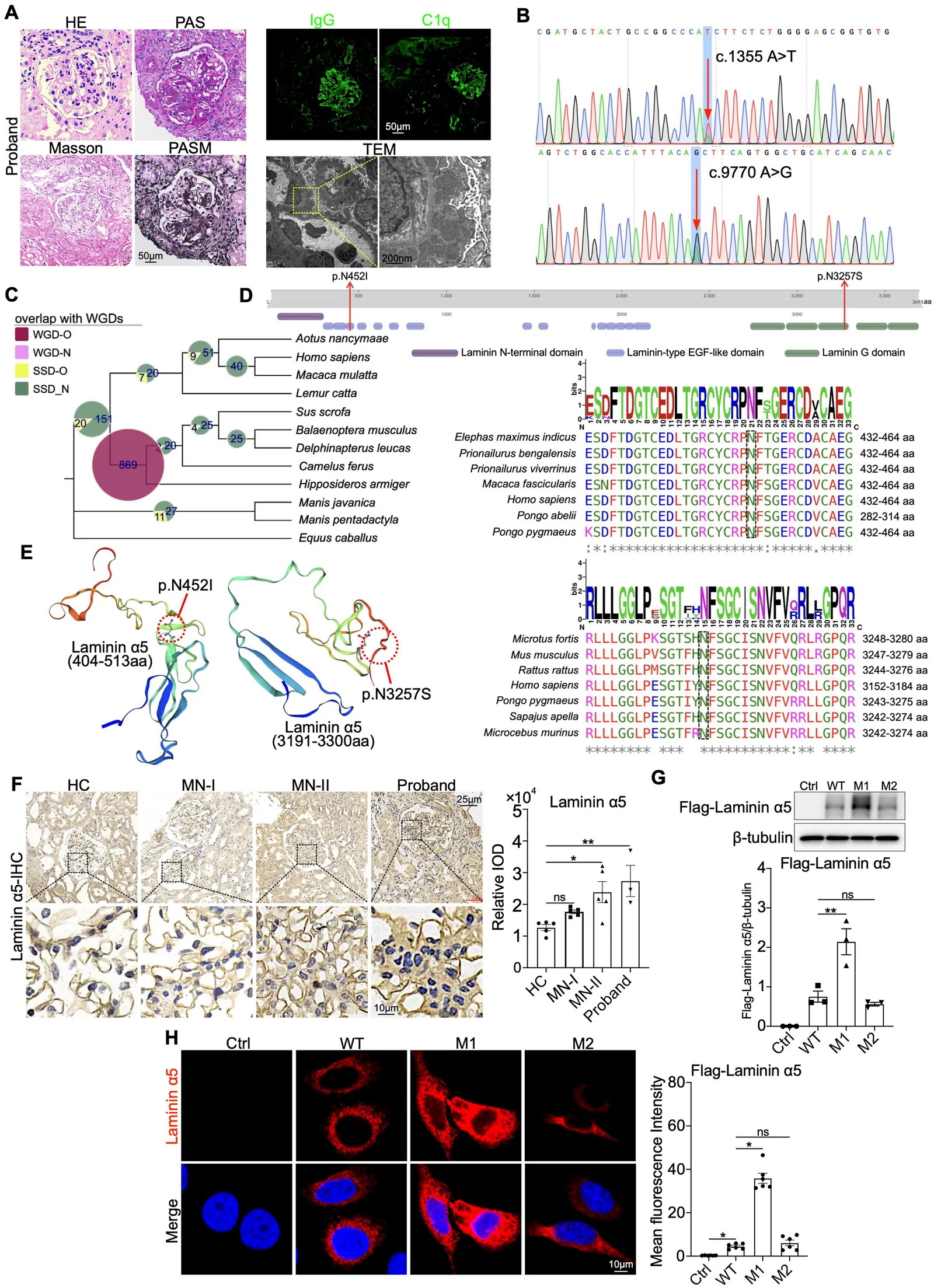
Correlations between proband clinical phenotypes and the compound heterozygous mutations in *LAMA5.***(A)** Light microscopy images of proband kidney biopsy samples, stained with various histochemical dyes (HE, PAS, PASM, Masson), reveal mesangial cell hyperplasia and abnormal glomerular basement membrane (GBM) thickness. Immunofluorescence staining detected deposition of IgG and C1q, while transmission electron microscopy observed the abnormal GBM thickness. **(B)** Exome sequencing identified the novel compound heterozygous mutations within the *LAMA5* gene (c.1355A>T and c.9770A>G) in the proband, confirmed by Sanger sequencing. **(C)** The phylogenetic tree of *LAMA5* indicated substantial conservation of the gene across various species. **(D)** Schematic of the Laminin α5 protein domains and ClustalW multiple sequence alignment of the amino acids at these mutation sites showed considerable conservation across diverse species. **(E)** Three-dimensional structural models of Laminin α5, from amino acids 404–513 and 3191–3300, respectively, predict folding and functional issues in the protein. **(F)** Immunohistochemistry showed elevated Laminin α5 expression in the glomerulus in the proband's kidney biopsy sample when compared with HC, MN-I, and MN-II. **(G, H)** Western blotting and immunofluorescence analysis of Laminin α5 variants' expression in HeLa cells transfected with transient-expression plasmids. HC, healthy control. MN-I/MN-II, stage I/II MN. ns, no significance. ∗*p* < 0.05, ∗∗*p* < 0.01, and ∗∗∗*p* < 0.001.

During our screening, we identified a pediatric patient (disease onset at age 5) harboring two novel heterozygous *LAMA5* variants: c.1355A>T (p.N452I; M1, maternal) and c.9770A>G (p.N3257S; M2, paternal). The patient presented with severe edema, oliguria, heavy proteinuria, hypoalbuminemia, and hyperlipidemia. Serologic testing for hepatitis B, ANCA, anti-GBM antibodies, and systemic autoantibodies was negative (Table 3). Renal biopsy revealed mesangial proliferation, GBM thickening, extensive podocyte foot process effacement, and widespread electron-dense deposits in subepithelial, subendothelial, mesangial, and intramembranous regions. Immunofluorescence showed focal deposition of IgG and complement C1q (Fig. 3A). Despite initial treatment with glucocorticoids and tacrolimus, proteinuria recurred, prompting genetic investigation.

**Table 3:** Clinical characteristics of the proband.

Characteristic	Items	Reference range	Results
Immunoglobulin and complement	cIgG (g/L)	5.28–21.9	6.3
	cIgA (g/L)	0.43–2.53	0.97
	cIgM (g/L)	0.48–2.26	4.27↑
	cIgE (IU/mL)	<150	16.2
	C3 (g/L)	0.7–2.06	0.53**↑**
	C4 (g/L)	0.11–0.61	0.11
Urinary parameters	24h urinary protein (g/24 h)	0–0.23	3.26↑
	RBC (cells/μL)	0–17	13
	Occult blood	–	2+
Autoantibody	Anti-ANA	–	–
	Anti-Sm	–	–
	Anti-dsDNA	–	–
	Anti-ANCA	–	–
	Anti-GBM	–	–
	Anti-PLA2R	<14 RU/mL	0.59
	Anti-THSD7A	105.5–365.5 ng/mL	130.79
Serum biochemical Parameters	Albumin (g/L)	38–55	22.7↓
	Cholesterol (mmol/L)	2.7–5.5	9.77↑
	Triglycerides (mmol/L)	0.3–1.8	2.63↑
	Urea (mmol/L)	2.2–7.14	5.2
	Creatinine (μmol/L)	14–60	28
	Uric acid	100–410	616↑
Concomitant disease	HBV infection	–	–
	Tumor	–	–
	Joint involvement	–	–

ANA, antinuclear antibody; dsDNA, double-stranded DNA; ANCA, anti-neutrophil cytoplasmic antibody.

Sanger sequencing confirmed both variants (Fig. 3B). Evolutionary conservation analysis indicated that both affected residues are highly conserved across species (Fig. 3C and D). Structural modeling predicted that M1 (located in the Laminin-type EGF-like domain) and M2 (within the Laminin G domain) could perturb protein conformation (Fig. 3E). Immunohistochemistry of the proband's renal tissue revealed markedly elevated Laminin α5 expression compared with healthy controls and age-matched MN patients (Fig. 3F). *In vitro* studies in HEK293T cells transfected with mutant constructs corroborated these observations: the M1 variant significantly increased Laminin α5 protein levels, whereas M2 had no discernible effect (Fig. 3G and H). Collectively, these data implicate the M1 (p.N452I) mutation as the primary driver of Laminin α5 overexpression and suggest a role for Laminin α5 dysfunction in autoimmune glomerular injury.

### The M1 (p.N452I) variant promotes GBM thickening via enhanced interaction with Collagen IV α3/α4/α5

**Figure 4:**
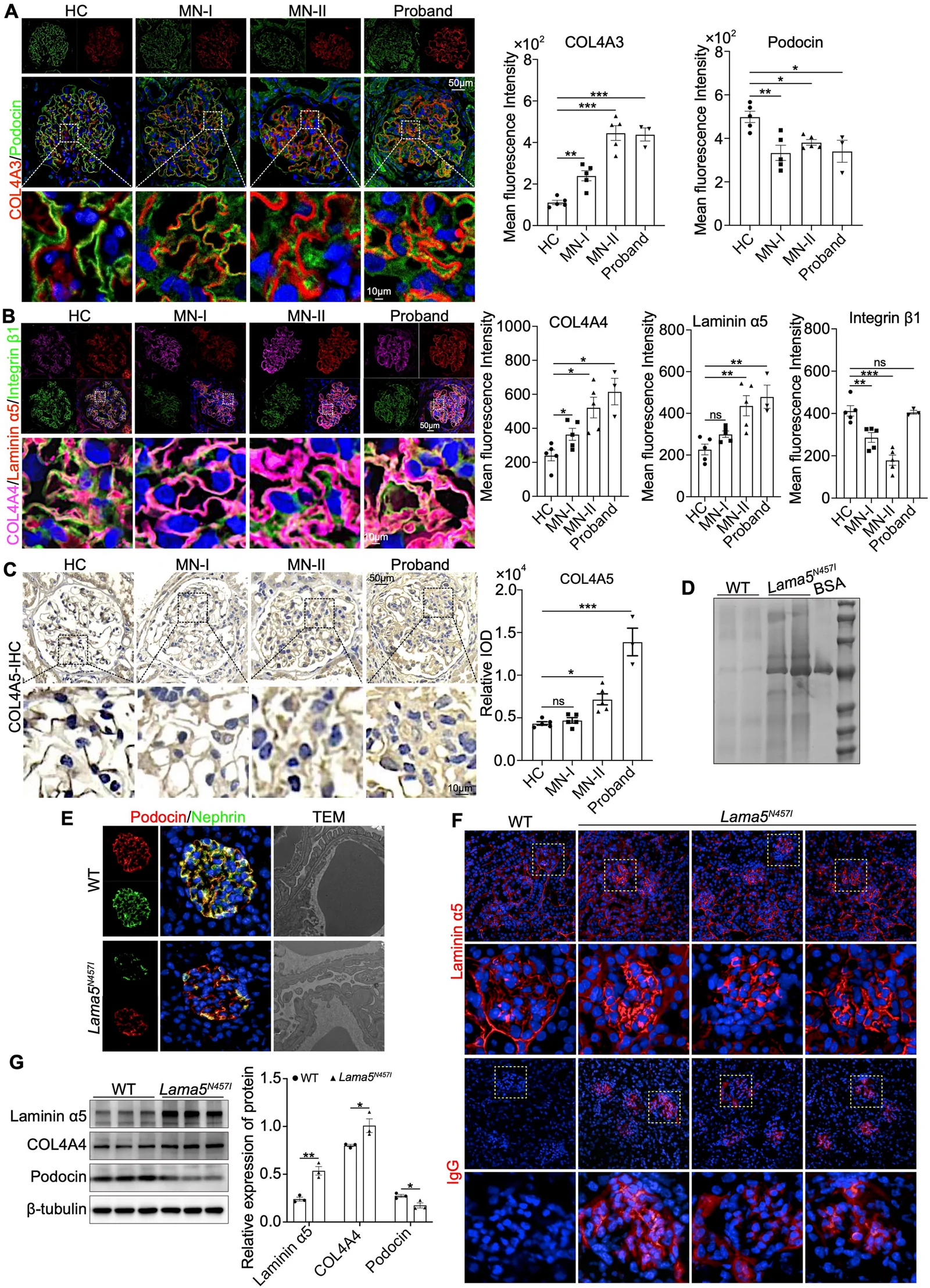
Enhanced interaction of Laminin α5 M1 variant with COL4A/3/4/5 linked to glomerular basement membrane (GBM) thickening. **(A)** Double-label immunofluorescence analysis investigated the linear structural expression of COL4A3 and Podocin in the GBM of the proband's kidney biopsy sample compared with HC, MN-I, and MN-II. **(B)** Three-label immunofluorescence comparative analysis explored the correlation between the linear structural expression of COL4A4, Laminin α5, and Integrin β1 in the aforementioned groups. **(C)** Immunohistochemistry analyzed the COL4A5 expression in the glomerulus in the proband's kidney biopsy sample when compared with HC, MN-I, and MN-II. **(D)** Coomassie brilliant blue staining assessed proteinuria in homozygous mutant mice. **(E)** Combined immunofluorescence and electron microscopy evaluation of glomerular pathology in homozygous mutant mice, including the expression of Nephrin and Podocin and structural changes in GBM and podocyte foot process. **(F)** Immunofluorescence staining for Laminin α5 expression and IgG deposition in glomeruli of homozygous mutant mice. **(G)** Western blotting evaluated the expression of Laminin α5, COL4A4, and Podocin in mutant mice. ∗*p* < 0.05, ∗∗*p* < 0.01, and ∗∗∗*p* < 0.001.

Given transmission electron microscopy evidence of pronounced GBM thickening in the proband and the essential role of Laminin α5 in GBM assembly, we sought to determine whether the *LAMA5* mutations directly contribute to this structural abnormality. Immunofluorescence analysis of the renal biopsy showed elevated linear expression of COL4A3, resembling the pattern seen in MN stage II (MN-II), alongside partial reductions in Podocin, Nephrin, and Synaptopodin (Fig. 4A; Fig. S2A). Further assessment revealed up-regulated linear expression of COL4A4 and Laminin α5, consistent with MN-II features, while Integrin β1 expression was variably decreased (Fig. 4B). Notably, COL4A5 expression was also significantly elevated in the proband compared with control groups (Fig. 4C). In contrast, Laminin β2 and γ1 showed markedly reduced expression (Fig. S2B). To model the functional impact of the p.N452I substitution *in vivo*, we generated a knock-in mouse harboring the orthologous *Lama5*^*N457I*^ mutation. Urinalysis using Coomassie Brilliant Blue staining revealed significant proteinuria in mutant mice (Fig. 4D). Immunofluorescence analysis demonstrated disrupted linear localization of Podocin and Nephrin, concomitant with intensified deposition of Laminin α5 and COL4A5 along the GBM. Strikingly, mutant mice also exhibited prominent glomerular IgG deposition, a hallmark of secondary MN, further supporting immune-mediated podocyte injury (Fig. 4E and F; Fig. S2C). These observations were corroborated by transmission electron microscopy, which showed marked GBM thickening and extensive podocyte foot process effacement (Fig. 4E). Western blotting analysis of kidney lysates confirmed elevated protein levels of Laminin α5 and COL4A4, alongside reduced Podocin expression, consistent with ongoing podocyte injury (Fig. 4G).

To determine whether the *LAMA5* variants directly up-regulate Collagen IV expression, we transiently transfected HeLa cells with wild-type (WT) or mutant *LAMA5* constructs. Western blotting and immunofluorescence analyses showed no increase in COL4A3/4/5 protein levels due to either mutant (Fig. S2D and S2E), suggesting that overexpression is not transcriptionally or translationally driven. We therefore hypothesized that the M1 variant might enhance physical interaction with Collagen IV networks. Co-immunoprecipitation assays revealed that the M1 mutant exhibited significantly stronger binding to COL4A3/4/5 compared with both WT and M2 (Fig. S2F). This gain-of-interaction phenotype provides a mechanistically coherent explanation for the pathological accumulation of extracellular matrix components and consequent GBM thickening observed in the proband.

### The M1 variant (p.N452I) in *LAMA5* drives the production of conformation-specific autoantibodies targeting laminin α5

**Figure 5:**
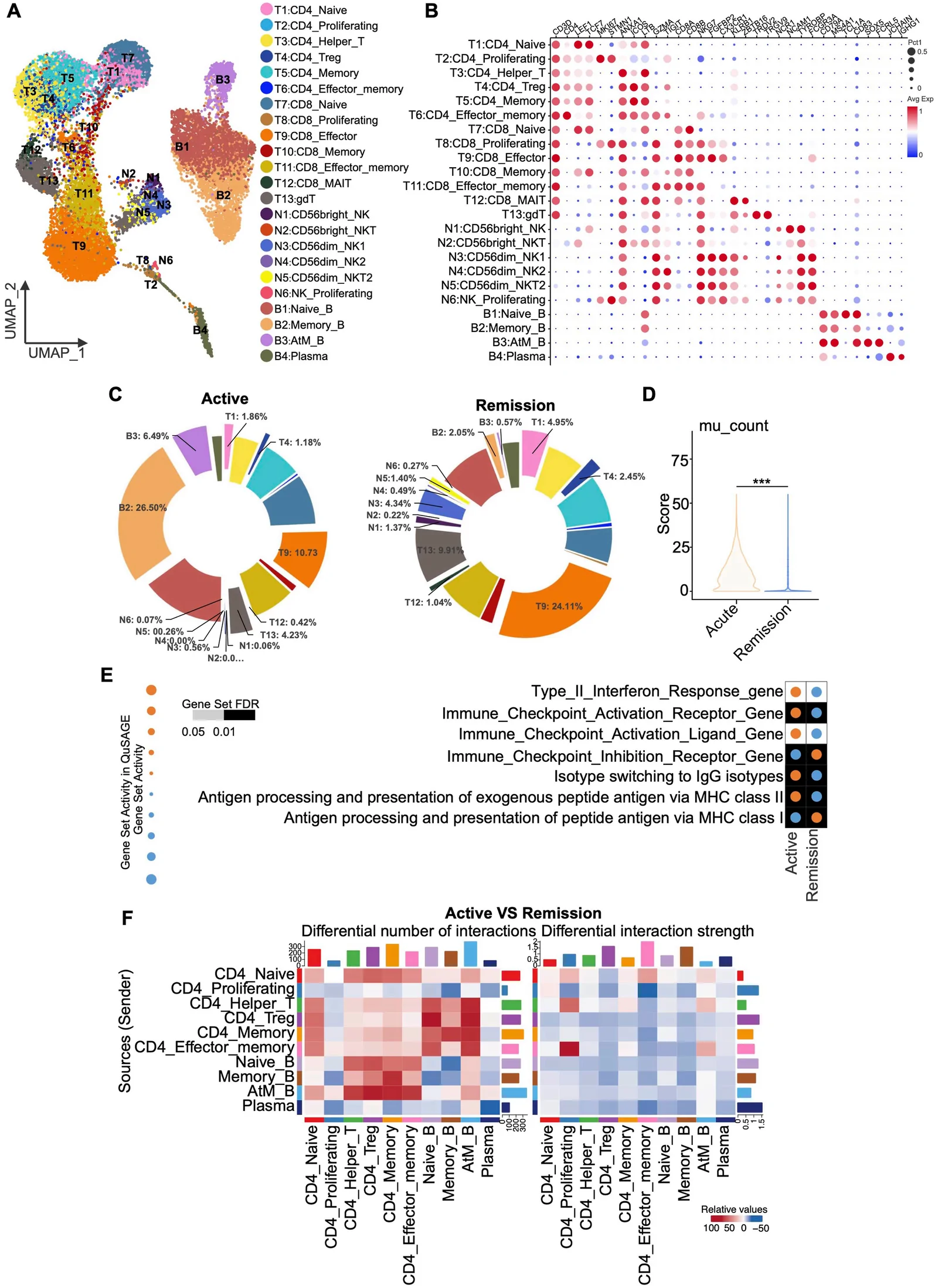
Single-cell sequencing suggested that the dysregulation of immune homeostasis in the proband drove the production of autoantibodies. **(A)** UMAP plot of lymphocytes derived from the blood of the proband in both the active and remission phases of proteinuria. Each dot represents an individual cell. **(B)** Bubble plots of the selected marker genes in 23 clusters. The size of each bubble indicates the proportion of cells within the indicated cluster that express detectable levels of genes, and the color depth of each bubble indicates the average expression level of genes. **(C)** Donut plots depicting the proportion of each cluster during the active and remission phases of the proband. Highlighted segments represent clusters with a > 2-fold change (active/remission ratio > 2 or < 0.5) between phases. **(D)** The differential analysis of somatic hypermutation rate of total B cells during the active and remission phase. The orange and blue represent active and remission, respectively. **(E)** QuSAGE analysis identifies several differentially expressed autoimmune-associated gene sets in B cells. The color depth of each bubble indicates the gene set activity, the white boxes indicate 0.01 ≤ FDR < 0.05, and the black boxes indicate an FDR < 0.01. **(F)** CellChat analysis identifies a differential number of interactions between CD4^+^ T cells and B cells. The red color (blue color) represents an increase (decrease) in the number or strength of interactions during the active phase versus remission.

To investigate the underlying immunological mechanisms, we performed single-cell RNA sequencing on peripheral blood lymphocytes collected during both active disease and remission. Integrated analysis of 17130 cells across 23 immune subsets revealed a marked (> 50%) reduction in multiple lymphocyte populations during active disease, including CD4^+^ naive T cells, regulatory T cells, CD8^+^ effector T cells, CD8^+^ mucosal-associated invariant T cells, γδ T cells, and all natural killer cell subsets (Fig. 5A–C and Table 4). This broad decline in these inhibitory and cytotoxic T lymphocytes indicates an impairment of immune tolerance. Conversely, among total B cells, the proportions of memory B and AtM B cells, a subset known for their autoreactive clonal expansion in chronic infections and autoimmune diseases, giving rise to antibody-secreting cell precursors, increased from 2.05% and 0.57% during remission to 26.50% and 6.49% during active disease, respectively (Fig. 5C and Table 4). Concurrent single-cell B cell receptor sequencing further demonstrated a significant rise in somatic hypermutation frequency and an elevated proportion of clonally expanded B cells (clonotype size > 7) in the active phase compared with remission (Fig. 5D; Fig. S3). Collectively, these B cell-related findings clearly indicate the activation of a B cell-driven humoral immune response in the proband. To further characterize B cell dysfunction, we applied Quantitative Set Analysis for Gene Expression (QuSAGE), which identified multiple differentially expressed gene sets enriched for autoimmune-related pathways in B cells during active disease (Fig. 5E). Additionally, CellChat-based intercellular communication analysis revealed an overall increase in interaction numbers between CD4^+^ T cells and various B cell subsets during active disease, along with enhanced interaction strength between T cells and AtM B cells, suggesting dysregulated T–B cell crosstalk that may promote autoreactivity (Fig. 5F).

**Table 4:**
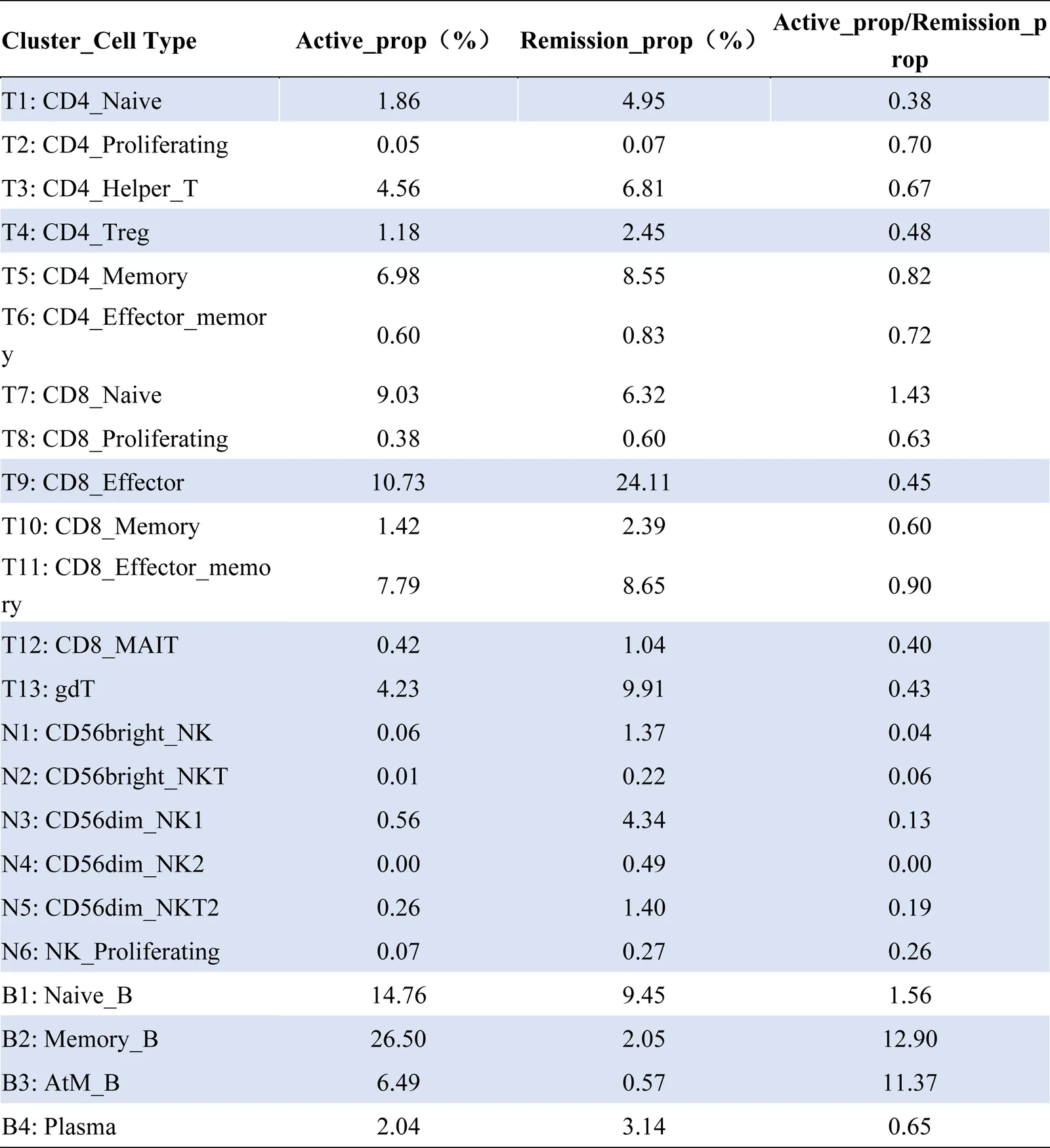
Comparison of lymphocyte subset proportions during the active and remission phases of proteinuria in the proband.

Rows with a > 2-fold change (active/remission) are highlighted in blue.

**Figure 6:**
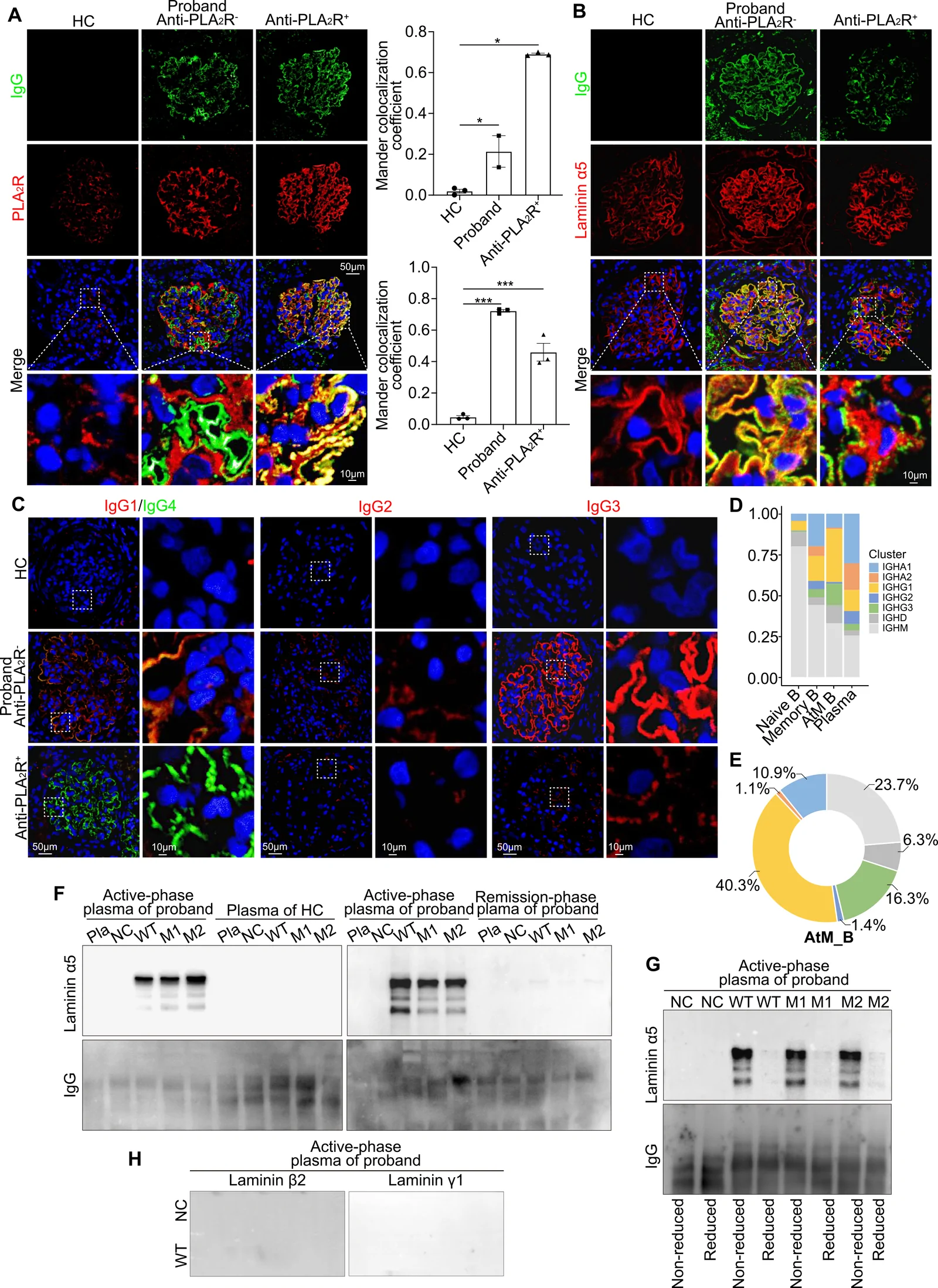
The *LAMA5* mutations resulted in the production and deposition of autoantibodies, IgG, targeting Laminin α5 within the glomerular basement membrane. **(A, B)** Double-label immunofluorescence comparative analysis investigated the protein localization relationship among PLA_2_R, Laminin α5, and IgG in both the proband and an Anti-PLA_2_R^+^ patient. **(C)** Immunofluorescence staining investigated the specific IgG subtypes present within the glomerulus of the proband. **(D, E)** Single-cell B cell receptor sequencing analyzed immunoglobulin heavy chain isotypes distribution in each B cell subset, and the donut plots show the distribution of immunoglobulin heavy chain isotypes in AtM B cells during the active phase of disease. **(F)** Immunoprecipitation analysis explored anti-Laminin α5 autoantibodies in the proband's plasma during both the active phase and remission phase. **(G, H)** Co-immunoprecipitation analysis of anti-Laminin α5 autoantibodies in the proband's plasma during the active phase assessed recognition of reduced and non-reduced Laminin α5, as well as Laminin β2 and Laminin γ1.

Given the spatial colocalization observed between anti-PLA_2_R IgG and PLA_2_R in PLA_2_R-positive MN, we examined whether IgG similarly colocalized with Laminin α5 in the proband's renal biopsy. Confocal immunofluorescence confirmed strong colocalization of IgG with Laminin α5 within the GBM, while no overlap was detected between IgG and PLA_2_R (Fig. 6A and B). To determine the IgG subclass composition of glomerular deposits and infer potential complement activation pathways, we performed subclass-specific immunofluorescence. Strikingly, unlike PLA_2_R^+^ MN patients, who typically show dominant IgG4 deposition, the proband exhibited predominant glomerular accumulation of IgG1 and IgG3 (Fig. 6C). This observation aligns with the single-cell B cell receptor sequencing results, which revealed that the immunoglobulin heavy chain genes in AtM B cells were predominantly IgHG1 and IgHG3 (Fig. 6D and E). To validate autoantibody specificity, we conducted Western blotting using plasma-derived eluates from both active and remission phases, probing against recombinant human Laminin α5 (wild-type, M1, and M2 variants). Autoantibodies against Laminin α5 were detectable only during active disease and were undetectable after rituximab treatment (Fig. 6F). Importantly, reactivity was observed exclusively with non-reduced Laminin α5 and was absent against reduced Laminin α5, Laminin β2, or Laminin γ1, confirming that the autoantibody is both conformation-dependent and α5-subunit-specific (Fig. 6G and H). Together, these results demonstrate that the M1 variant (p.N452I) could induce the production of conformation-specific, self-reactive IgG antibodies targeting Laminin α5, which deposit in the GBM and likely contribute to autoimmune glomerular injury.

### Subtyping of IgG showed dominant IgG1 and IgG3 within the glomerulus of the *LAMA5* proband

**Figure 7:**
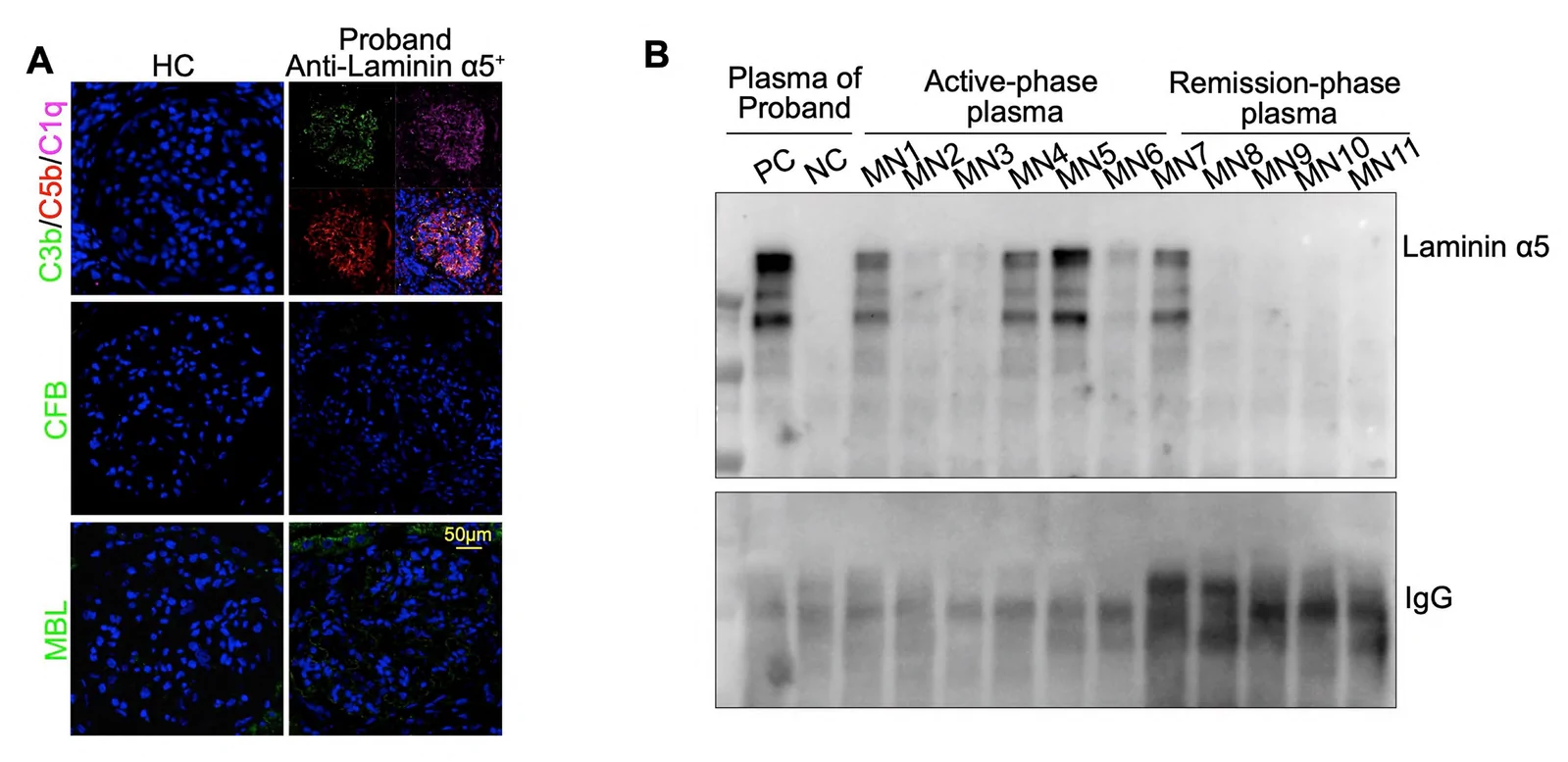
Anti-Laminin α5 autoantibodies initiated activation of the classical complement pathway within the glomeruli and had a certain clinical value in the immunotherapy of membranous nephropathy. **(A)** Immunofluorescence staining determined which complement pathway was active within the glomerulus of the proband. **(B)** Immunoprecipitation analysis explored whether anti-Laminin α5 autoantibodies existed in membranous nephropathy patients without *LAMA5* mutation during both the active phase and remission phase. PC, positive control; NC, negative control; ∗∗∗*p* < 0.001.

The predominant subclass in deposits of PLA_2_R^+^ and THSD7A^+^ MN is IgG4, displaying notable glomerular deposition of complement components, including C1q, the initiator of the classical pathway of complement, in kidney biopsies from patients with PLA_2_R^+^ and THSD7A^+^ associated MN.^19^^,^^20^ Subsequently, a detailed examination of key molecules involved in the three pathways was conducted to determine the activated signaling pathway. The results demonstrated that deposits of IgG1 and IgG3 primarily activated the classical pathway, supported by the expression of C1q, C3b, and C5b (Fig. 7A). Importantly, there was an absence of expression of CFB, the initiating protein of the alternative pathway, and MBL, the initiating protein of the lectin pathway (Fig. 7A). These findings indicated that the novel *LAMA5* mutations resulted in the deposition of self-specific antibodies, particularly IgG1 and IgG3, within glomeruli, triggering the activation of classical signaling pathways.

Given this evidence of autoantibody-mediated injury, we next investigated whether anti-Laminin α5 autoantibodies might also be present in children with MN who do not carry *LAMA5* mutations. We therefore analyzed plasma samples from pediatric MN patients during both the active phase and the remission phase after treatment, specifically testing for circulating autoantibodies against Laminin α5 (Fig. 7B and Table 5). Our results revealed that there may be an association between the presence of anti-Laminin α5 autoantibodies during active disease and clinical response to rituximab therapy. Among patients who achieved remission following rituximab treatment, including the proband, anti-Laminin α5 autoantibodies were detectable in plasma during the active phase. Meanwhile, the patient who entered remission without receiving rituximab did not have detectable anti-Laminin α5 autoantibodies in the plasma. Of note, with detectable anti-laminin α5 IgG in the remission phase, patient MN7 experienced a rapid relapse following rituximab therapy, suggesting a poor response to the treatment. These observations may suggest that anti-Laminin α5 autoantibodies may define a distinct subset of MN that responds to B-cell-targeted therapy. Furthermore, testing for these autoantibodies during the active phase could serve as a potential biomarker to guide the use of rituximab in pediatric MN patients.

**Table 5:** Clinical characteristics of membranous nephropathy. Supportive treatment refers to treatment without any steroids or immunosuppressants.

Index patient	Age of onset (yr)	Time to onset (m)	Sex	Anti-PLA_2_R antibody	Proteinuria (g/24h)	eGFR	IF Microscopy	Therapy pre-detection	Pathologic stage	Clinical Phase at detection	1-year outcome post-RTX treatment
IMN1	5.58	52.00	Female	–	0.36	109.53	IgG (++), IgA(+/−), IgM(−), C3 (−), C1q (++)	Steroids + TAC	II	Active	/
IMN2	11.17	0.80	Female	–	4.05	188.84	IgG (+++), IgA(−), IgM(+/−), C3 (+++), C1q (−)	Steroids + RTX	II	Active	No relapse
IMN3	14.92	3.00	Male	–	0.90	97.555	IgG (+++), IgA(−), IgM(+), C3 (−), C1q (−)	Steroids + RTX	II	Active	No relapse
IMN4	3.67	1.00	Male	+-	0.83	105.36	IgG (++), IgA(−), IgM(+), C3 (+/−), C1q (+/−)	Supportive treatment	II	Active	No relapse
IMN5	13.08	0.60	Male	+	4.27	171.81	IgG (+++), IgA(−), IgM(+), C3 (++), C1q (−)	Steroids	II	Active	No relapse
IMN6	10.08	4.80	Female	–	5.67	131.06	IgG (++), IgA(−), IgM(+), C3 (+), C1q (−)	Steroids + TAC	I ∼ II	Active	/
IMN7	6.08	18.80	Male	–	0.01	116.14	IgG (+++), IgA(−), IgM(+), C3 (++), C1q (−)	Steroids + TAC	I	Remission	Relapse
IMN8	12.08	21.00	Female	–	0.02	89.67	IgG (++), IgA(−), IgM(+), C3 (+/−), C1q (−)	Steroids + TAC	I ∼ II	Remission	/
IMN9	12.42	14.50	Male	–	0.06	115.97	IgG (+++), IgA(−), IgM(+), C3 (−), C1q (−)	Supportive treatment	IV	Remission	/
IMN10	10.58	20.20	Male	–	0.07	144.99	IgG (+++), IgA(−), IgM(+), C3 (+++), C1q (−)	Steroids + TAC	III	Remission	/
IMN11	16.58	19.00	Male	+	0.05	117.56	IgG (+++)IgA(+); IgM(+); C3 (++); C1q (−)	Supportive treatment	III	Remission	/

Note:”/” indicates patient lost to follow-up or not treated with RTX. TAC, tacrolimus; RTX, rituximab.

## Discussion

MN, characterized by GBM thickening, subepithelial immune deposits, and heavy proteinuria, remains a leading cause of nephrotic syndrome.^21^^,^^22^ While autoimmune forms driven by PLA_2_R or THSD7A autoantibodies are well established, a substantial proportion of pediatric MN cases lack known autoantigens, suggesting alternative pathogenic mechanisms. Here, we identify compound heterozygous mutations in *LAMA5*, which encodes the α5 chain of Laminin α5β2γ1, particularly the p.N452I (M1) variant, as a novel genetic driver of autoimmune glomerular injury that phenocopies secondary MN. Unlike COL4A3/4/5 mutations in Alport syndrome, which disrupt GBM ultrastructure without inducing IgG deposition, the M1 variant triggers both structural and immune-mediated injury. Located within a laminin-type EGF-like domain, the M1 variant causes conformational dysregulation of laminin α5, leading to its overexpression and enhanced binding to COL4A3/4/5. This gain-of-interaction phenotype drives pathological GBM thickening and matrix accumulation, recapitulated in patient biopsies and *Lama5*^*N457I*^ knock-in mice that developed proteinuria, podocyte effacement, and glomerular IgG deposits. Critically, the M1-induced structural alteration breaks immune tolerance, eliciting conformation-specific autoantibodies, predominantly of the IgG1 and IgG3 subclasses. These autoantibodies colocalize with Laminin α5 in the GBM, activate the classical complement pathway, and disappear upon rituximab-induced clinical remission. Their presence during active disease and correlation with treatment response define a distinct, B-cell-responsive MN endotype. Single-cell immune profiling further supports an antigen-driven process, revealing clonal expansion of autoreactive B cells, deficiency in suppression of autoimmunity, and disrupted T–B cell communication during active disease. Importantly, this condition is distinct from anti-GBM disease (Goodpasture syndrome). Patients show no pulmonary involvement, no linear IgG along alveolar basement membranes, and no reactivity against the non-collagenous domain of Collagen IV α3, the canonical target in Goodpasture.^23^, ^24^, ^25^, ^26^ Thus, Laminin α5-associated MN represents a separate autoimmune entity.

Beyond its structural role in the GBM, Laminin α5 exerts significant immunomodulatory functions, including shaping lymph node architecture, modulating chemokine expression, influencing T cell migration and differentiation, and supporting marginal zone B cell survival, all of which contribute to the regulation of humoral immunity.^27^, ^28^, ^29^ Our findings demonstrate that the M1 variant not only perturbs GBM integrity but also serves as a neo-autoantigen, triggering an antigen-specific autoimmune response. The detection of anti-Laminin α5 autoantibodies in a subset of MN patients during active disease, coupled with favorable responses to rituximab, underscores their pathogenic relevance. These autoantibodies differ fundamentally from those in classic anti-GBM disease: they arise secondarily to a conformational change induced by a *LAMA5* gain-of-function mutation, rather than through direct targeting of native GBM components.^30^^,^^31^ Although the precise genetic and immunological interplay remains incompletely understood, the potential overlap between Laminin α5 dysfunction and anti-GBM pathology warrants ongoing clinical vigilance. Moreover, Laminin α5 influences B-cell fate by supporting marginal zone B-cell survival and modulating antibody responses.^32^^,^^33^ Our data also reveal discrepancies in the linear expressions of Laminin α5, β2, and γ1, suggesting that *LAMA5* mutations may impact the GBM's component ratio. Furthermore, Laminin α5's interaction with Integrin α3β1 in podocytes underscores its role in cellular integrity and function.^34^^,^^35^ Laminin's binding to type IV Collagen highlights the intricate molecular interactions within basement membranes, which may contribute to the up-regulation of COL4A3/4/5 and Integrin β1 expression.^36^ These findings illuminate the complex interplay between Laminin, Integrins, and Collagen in MN pathogenesis.

From an immunopathological perspective, approximately 75% of idiopathic MN patients have anti-PLA_2_R antibodies, and 1%–3% have anti-THSD7A antibodies, with IgG4 predominating. IgG4 does not activate C1q but can trigger the lectin pathway via mannan-binding lectin in a glycosylation-dependent manner.^37^^,^^38^ In contrast, the child with *LAMA5*-mutant MN exhibited glomerular deposition of IgG1 and IgG3, which directly activate the classical complement pathway via Fc–C1q interaction, promoting membrane attack complex formation and podocyte injury.^39^ Since 2014, multiple novel MN antigens (*e.g.*, EXT1/2, NELL1, SEMA3B, NCAM-1) have been reported at low frequency in pediatric MN, usually associated with secondary MN.^39^ Among these, SEMA3B is most strongly linked to childhood MN, with IgG1 autoantibodies depositing along the tubular basement membrane.^40^ Laminin α5, by contrast, appears directly linked to a genetic variant and simultaneously induces GBM thickening and autoantibody production in the absence of systemic disease or tumors.

This study has limitations. Mechanistic insights are primarily based on a single case, which limits generalizability. Although the key mutation site was characterized *in vitro*, the immunological mechanisms driving autoantibody generation remain incompletely defined, particularly *in vivo*. Preliminary screening for anti-Laminin α5 antibodies in a small pediatric cohort was constrained by sample size and clinical testing capacity, preventing comprehensive evaluation of genomic and renal tissue correlations. Future studies with larger cohorts are required to validate these findings and explore the prevalence of Laminin α5-associated MN.

## Conclusions

Our study demonstrates that specific mutations in the *LAMA5* gene, including the novel p.N452I variant, drive a unique genetic-immunological pathway in GBM pathology that is distinct from mechanisms involving Collagen IV. This mutation leads to GBM thickening and loss of its normal linear architecture by promoting pathological aggregation of Collagen IV. At the same time, it disrupts immune homeostasis, resulting in the production of autoantibodies against Laminin α5. These autoantibodies specifically target glomerular Laminin α5, activate the classical complement pathway, and cause cellular damage. Importantly, fluctuations in circulating levels of anti-Laminin α5 antibodies may serve as a predictive biomarker for evaluating the effectiveness of immunotherapy in pediatric patients with MN. Together, these findings uncover a new disease mechanism linking a defined genetic alteration to both structural and immune-mediated kidney injury, offering significant potential for improved diagnosis, monitoring, and targeted treatment in this patient population.

## CRediT authorship contribution statement

**Han Xiao:** Writing – original draft, Project administration, Methodology, Funding acquisition. **Hui Yin:** Project administration, Methodology, Formal analysis. **Yulu Shi:** Resources, Investigation. **Xindi Zhou:** Project administration, Methodology. **Qinqin Tan:** Methodology. **Lan Qiu:** Investigation. **Chenyang Xiong:** Investigation. **Wanbing Chen:** Methodology. **Xuejun Yang:** Resources. **Wei Jiang:** Writing – review & editing, Writing – original draft, Funding acquisition, Conceptualization. **Haiping Yang:** Writing – review & editing, Resources, Formal analysis, Conceptualization. **Qiu Li:** Writing – review & editing, Funding acquisition, Conceptualization.

## Ethics declaration

The study obtained informed consent from patients at stage III before their participation in the research. The research protocol received approval from the Ethics Committee of the Children's Hospital of Chongqing Medical University, with the assigned Approval No. (2023) Ethics Review (Research) No. 304. Written informed consents were obtained from patients for publication of the information contained within this article.

## Data availability

All data and materials included in this study are available upon request by contacting the corresponding author.

## Funding

This study was supported by the 10.13039/501100001809National Natural Science Foundation of China (No. U24A20668), the Joint Medical Research Programs of Chongqing Science and Technology Bureau and Health Commission Foundation (China) (No. 2023GGXM001), the Chongqing Natural Science Foundation (China) (No. CSTB2025NSCQ-GPX1191), the Chongqing Science and Health Joint Medical Research Project (China) (No. 2026QNXM056), the 10.13039/501100002858China Postdoctoral Science Foundation (No. 2024MD764055), and the Western China Birth Cohort (No. NCRCCHD-2023-MP-01).

## Conflict of interests

The authors declared no conflict of interests.

## Figures and Tables

**figs1:**
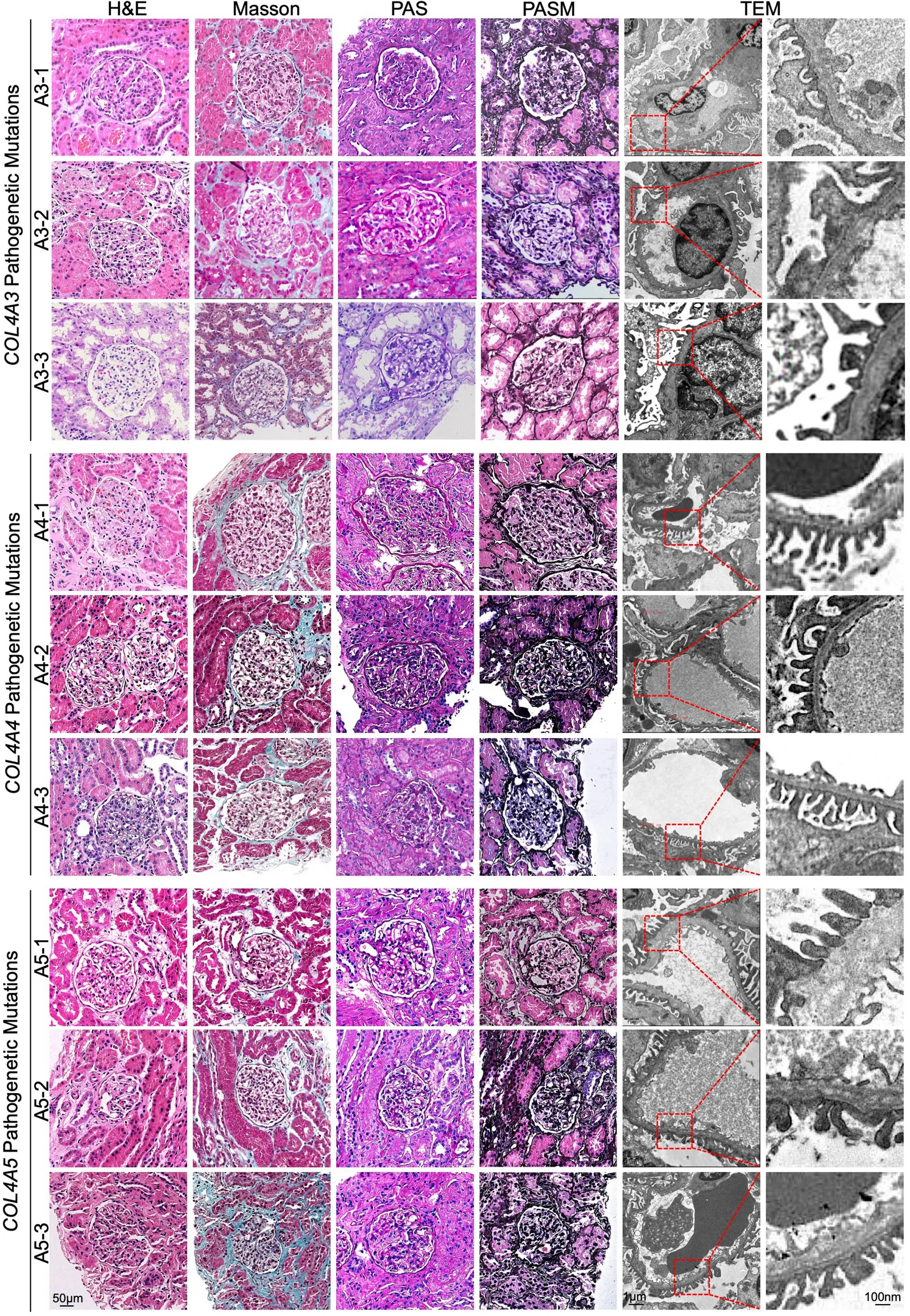


**figs2:**
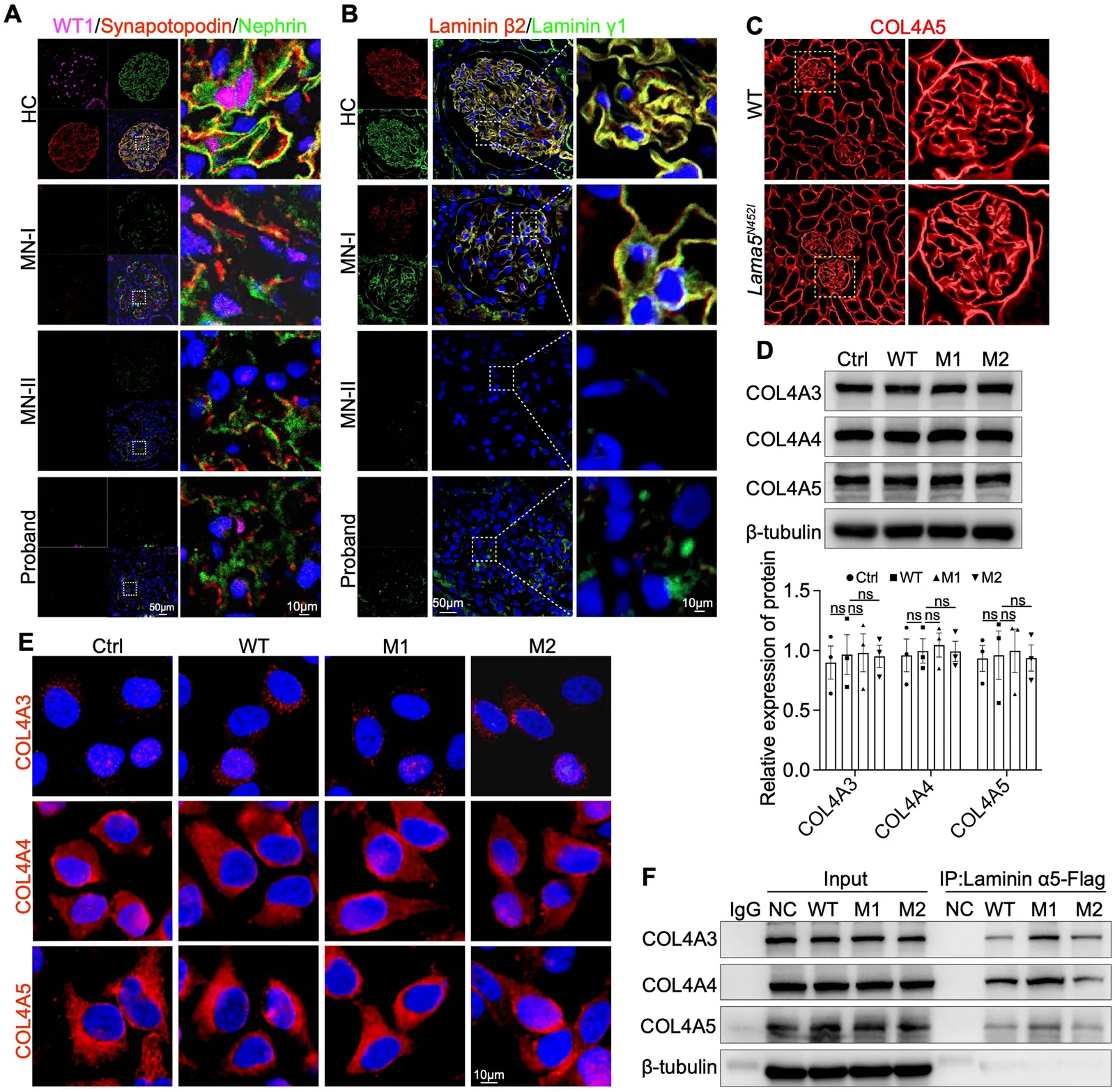


**figs3:**