# Supplementary material for: LAMA5 pathogenic variant uncovers a novel autoantigen in membranous nephropathy
Source: Genes Dis. 2026 Jun 13;14(1):102301. doi: 10.1016/j.gendis.2026.102301 (PMC13589091; doi:10.1016/j.gendis.2026.102301)
Supplement: Supplementary file 1 [file mmc1.docx]

**Figure S1** Examination of renal histopathological alterations in individuals with Alport syndrome induced by mutations in *COL4A3*, *COL4A4*, or *COL4A5* genes. Histological assessments using HE, PAS, PASM, and Masson staining revealed a spectrum of pathological features, including mesangial cell proliferation, inflammatory cell infiltration, fibrosis, glomerular sclerosis, and segmental sclerosis. Transmission electron microscopy further elucidated variations in glomerular basement membrane thickness and podocyte foot morphology.

**Figure S2** Comparative analysis of the effects of Laminin α5 M1 variants on the expression of glomerular basement membrane-related molecules and podocyte markers. **(A)** Three-label immunofluorescence analysis investigated the linear structural expression of Synapotopodin and Nephrin and the expression of WT1 in the glomerulus of the proband's kidney biopsy sample compared with HC, MN-I, and MN-II. **(B)** Double-label immunofluorescence analysis investigated the linear structural expression of Laminin β2 and Laminin γ1 in the aforementioned groups. **(C)** Immunofluorescence staining investigated the expression of COL4A5 in glomeruli of mutant mice. **(D)** Western blotting assessed the impact of Laminin α5 variants on the expression of COL4A3/4/5. **(E)** Assessment of Laminin α5 variants' expression in HeLa cells transfected with transient expression plasmids and its impact on the expression of COL4A3/4/5 through immunofluorescence staining analysis. **(F)** Evaluation of the impact of Laminin α5 variants on their binding ability with COL4A3/4/5 through co-immunoprecipitation analysis. ns, no significance.

**Figure S3** Distribution of clonally expanded B cells, and the color-coded by clonal size: gray (<2), red (2–7), and green (>7).
